# Establishing One Health Surveillance Platform for Electronic Integrated Disease Surveillance and Response in Malawi: Action Design Research Study

**DOI:** 10.2196/72029

**Published:** 2026-01-26

**Authors:** Tsung-Shu Joseph Wu, Matthew Vundu Mvula, Edward Kada Koma Chado, Blessings Nthezemu Kamanga, Daniel Denis Mapemba, Rajab Enoch Billy, Louis Nyirongo, Annie Chauma Mwale, Evelyn Chitsa Banda, Matthew Kagoli, Tiwonge Davis Manda, Gunnar Aksel Bjune, Jens Johan Kaasbøll

**Affiliations:** 1 Department of Informatics Faculty of Mathematics and Natural Sciences University of Oslo Oslo Norway; 2 Research Department Luke International Oslo Norway; 3 Health Information Systems Department Luke International Lilongwe Malawi; 4 Overseas Mission Department Pingtung Christian Hospital Pingtung City Taiwan; 5 Department of Epidemiology and Surveillance Public Health Institute of Malawi Lilongwe Malawi; 6 National Public Health Research Division Public Health Institute of Malawi Lilongwe Malawi; 7 Department of Computing University of Malawi Zomba Malawi; 8 Institute for Health and Society Faculty of Medicine University of Oslo Oslo Norway

**Keywords:** adaptive digital health system, DHIS2, District Health Information Software, eIDSR, IDSR, integrated disease surveillance and response, One Health surveillance, One Health, pandemic preparedness

## Abstract

**Background:**

Facing the threats of emerging and reemerging health issues requires One Health surveillance systems to provide information for integrated responses. Malawi started enhancing the electronic integrated disease surveillance and response (eIDSR) system in 2015, progressing with the aim of developing a One Health Surveillance Platform (OHSP) using District Health Information Software 2 (DHIS2) as its technical backbone, thereby supporting the COVID-19 pandemic response more resiliently and impacting the integrated disease surveillance and response (IDSR) performance. Digital solutions are critical components of One Health surveillance; however, evidence of the successful establishment and implementation of adaptive digital One Health surveillance systems is scarce.

**Objective:**

This study aims to report on the establishment of the OHSP in Malawi and how an adaptive digital health solution contributed to strengthening and impacting the country’s eIDSR during the COVID-19 pandemic and beyond the pandemic.

**Methods:**

The establishment of Malawi’s OHSP was based on the action design research methodology with a transdisciplinary approach. The core team reflected the multiple iterative processes of building the OHSP and formalized its impact on IDSR reporting quality.

**Results:**

The OHSP core team conducted multiple iterative cycles to build the platform, leveraging lessons from previous eIDSR pilots, reusing digital health infrastructure, and developing DHIS2 digital solutions in 2019, right before the COVID-19 pandemic. The initial establishment was to cover 48.3% (14/29) of the country’s health districts. Pivoting from the initial plan as the COVID-19 pandemic emerged, the core team swiftly adapted the OHSP to scale up nationwide and assisted the health system in responding to the pandemic. The pandemic shock resulted in a national scale-up of the OHSP and impacted the national weekly IDSR reporting quality from nonexistence in 2015 to 97.8% and 74.5% for completeness and timeliness, respectively, in 2024.

**Conclusions:**

The establishment of the OHSP significantly bolstered the surveillance function for weekly IDSR reporting. Government leadership and good coordination were key to success. Continuous capacity building, enhancement of community-level surveillance with digital innovations, adaptable technical infrastructure, and a reuse strategy can provide long-term sustainability for One Health surveillance. Malawi’s experience may apply to other countries with demonstrated value of resilient, government-led digital health interventions. Future efforts should focus on improving interoperability with other One Health domains and investing in infrastructure upgrades with local leadership and domestic funding to prepare for future emergencies.

## Introduction

Emerging and reemerging health threats, coupled with the complexity of modern health systems, underscore the need for an integrated approach to health surveillance and response, known as the One Health approach [1-3]. Leveraging the One Health approach, the One Health surveillance system emphasizes intersectoral and multidisciplinary collaboration and integrated surveillance of human, animal, and environmental health, which offers promising avenues for strengthening public health management and pandemic preparedness [2,3]. The recent COVID-19 pandemic highlights the critical need to adopt and realize the One Health approach–aided surveillance systems to foster the health system’s resilience and reduce disease risks [1,3]. However, given the broad scope of One Health, a proper implementation model for examining the One Health surveillance system and resilient health systems has yet to be fully developed [1,4].

In strengthening health system resilience, surveillance is essential for enhancing the health system’s governance function during the shock onset, alert, and impact management stage [5]. Considering health system resilience and recent COVID-19 pandemic experience, effective information and communication systems and digital technologies–empowered surveillance can aid health authorities in responding more resiliently [5-7]. The “One Health surveillance system” is considered a collaborative surveillance system that provides the systematic collection, validation, analysis, and interpretation of data, and dissemination of information collected from humans, animals, and the environment to inform decisions for more effective, evidence-based health interventions [2,8]. By linking surveillance data across different sectors and applying a transdisciplinary approach, One Health surveillance can help different health-related actors address the full spectrum of disease and outbreak detection, emergency preparedness, response, management, and global health security and health system resilience [2,4].

However, establishing, realizing, and implementing a functional One Health surveillance system has proven challenging, with findings showing that the absence of a clear vision for the future of One Health is a barrier to interdisciplinary collaboration and that siloed approaches by different sectors restrict the ability of professionals to work collaboratively across disciplines [9,10]. At the policy level, political will and legal basis are essential for integrating surveillance systems yet remain difficult to attain the collaborative goal [2,8,10]. At the institutional level, establishing a formal governance body with representatives from each sector could assist in overcoming long-standing barriers of privacy and distrust, where sociopolitical factors were considered as organizational attributes contributing to establishing a One Health surveillance system [2,8-10]. At the operational level, technical barriers highlight that the requirements for a successful One Health surveillance system should include understanding disease transmission and the importance of domain experts’ knowledge as scientific support, as well as technical mechanisms to support collaboration [2,10].

Malawi adopted the integrated disease surveillance and response (IDSR) system as its national surveillance system for human health and recognized the need for improvements through advanced information technologies [11]. The latest IDSR technical guidelines highlight the importance of using electronic and digital tools to strengthen its function, known as electronic integrated disease surveillance and response (eIDSR) [12]. Countries are urged to implement electronic tools with an interoperable approach to fortify the eIDSR system and information-sharing platforms between various health units to expedite data transmission and enable a swift response to public health threats [12].

In 2014, the Ministry of Health (MoH) sought to enhance the nation’s health emergency preparedness plan and piloted an in-house–developed eIDSR system for the first time [13]. After the World Bank–funded eIDSR pilot project, the Public Health Institute of Malawi (PHIM) of the MoH carried out another pilot by using SMS text message technology—Argus—to aid periodic eIDSR reporting [14]. However, these 2 pilots could not be sustained and scaled up. Meanwhile, Malawi recognized the importance of adopting a One Health approach and leveraging digital tools for surveillance by strengthening the eIDSR system as a starting point for the human health domain in the country [11,15,16].

After Cyclone Idai in 2019, the postdisaster needs assessment revealed the urgent need for strengthening and sustaining epidemiological surveillance and emergency preparedness for overall health system resilience [17]. As Malawi has responded continuously to numerous health disasters, including floods, droughts, and disease outbreaks, the idea of the One Health Surveillance Platform (OHSP) originated amidst the pilots of eIDSR, a successful response to an animal-originated anthrax outbreak and the postdisaster period learning [18]. As a result, the MoH chose the District Health Information Software 2 (DHIS2), a technology that has been used for Malawi’s health management information system (HMIS) with a successful nationwide scale-up [16,19], as the technical backbone to establish the OHSP.

Health system resilience is the ability of the system to prepare for and respond to sudden shocks and everyday challenges, and its capacity to absorb deteriorations, adapt, and transform to cope with them [5,20]. It requires strong leadership, political will, a collaborative and coordinated governance structure, and robust surveillance systems to detect shocks and their impact in a timely manner [5,6]. In response to the emerging challenges posed by the COVID-19 pandemic, the MoH decided to accelerate the speed of the establishment of the OHSP to accommodate emerging needs. Malawi was able to use the OHSP as one of the digital health innovations, thereby bouncing forward resiliently during the COVID-19 pandemic [21]. This study aims to report on the establishment of OHSP in Malawi and how an adaptive digital health solution contributed to strengthening and impacting the country’s eIDSR during the COVID-19 pandemic and beyond the pandemic.

## Methods

### Overview

The establishment of Malawi’s OHSP for eIDSR started in 2015 based on the action design research (ADR) methodology [22] with the initial intention to strengthen the country’s IDSR system. The ADR method has four stages: (1) problem formulation; (2) building, intervention, and evaluation (BIE); (3) reflection and learning; and (4) formalization of learning [22]. We chose this method to ensure that practical insights, stakeholder feedback, and emerging needs were continually incorporated into the platform’s development.

### Context of the Public Health Surveillance System in Malawi

Malawi is a landlocked country situated in southeastern Africa with a population of almost 16 million. It borders Tanzania, Zambia, and Mozambique [23]. Administratively, the country is divided into 3 regions—northern, central, and southern—with further demarcation into 5 zones, 29 health districts, and 4 major cities. The epidemiology department of the PHIM within the MoH is the main custodian of the IDSR system.

Malawi adopted the IDSR strategy in 2002, and the third edition technical guidelines were published in May 2014 with incremental notifiable diseases and health conditions to fulfill the International Health Regulations (IHR) 2005 and public health needs [24]. Malawi conducted a nationwide assessment of the IDSR system in 2017 [11] and found that the IDSR system showed relatively good completeness but poor timeliness of monthly reports nationwide and zero weekly reports. The use of IT to overcome challenges and improve the surveillance system to have better timeliness for IDSR reporting was proposed [11]. Ever since the assessment, PHIM has been working on strengthening the nation’s IDSR system activities with electronic and digital solutions, and later on added the One Health surveillance vision after successfully responding to a hippopotamus’ original anthrax outbreak in 2019 [18].

### Research Materials and Analysis Methods

Our analysis centered around various documents, including policies, meeting minutes from focus group meetings and key informant interviews, field notes, technical documentation, and digital health products used during the establishment of the OHSP. The OHSP is a digital health innovation accelerated during the COVID-19 pandemic for resilient responses [21] and was sustained for eIDSR functions beyond the pandemic. We summarize the findings from the current establishment to fill the existing knowledge gaps in the implementation of the digitalized OHSP. The impact of the OHSP digital health innovations on the weekly IDSR reporting quality was analyzed using 2024 weekly IDSR reporting quality data with the built-in function of the Reporting Rate Summary in the DHIS2, using the same approach as applied in the previous study [11].

### Reflection and Learning Analytic Tool for OSHP and Impact on IDSR System

In order to rigorously reflect on how the OHSP establishment, and the use in responding to the COVID-19 pandemic resiliently, an analytic tool against the resilience framework was synthesized from the available literature, covering the One Health surveillance systems establishment, attributes of engineering resilient information systems for emergency response, strengthening health systems resilience, and known challenges of implementing DHIS2 [2,5,6,9,10,25,26]. Based on the synthesized findings, we first defined the following five analysis domains: (1) One Health surveillance characteristics, (2) addressing known DHIS2 challenges, (3) health system resilience phases, (4) health system resilience attributes, and (5) health system resilience tools [2,5,6,25]. We then identified thematic areas [2,5,6,25,26] and their corresponding establishment level of a One Health surveillance system, namely policy, institutional, and operational levels, based on Bordier et al [2]. Key attributes and challenges were identified from the reviewed literature [1,2,5,6,9,10,25-28]. We selected the relevant strategies and principles applied to the thematic areas and finally selected attributes for our analysis. The high-level analytic tool is shown in Table 1, and the indicators are detailed in Multimedia Appendix 1 [1,2,5,6,9,10,25,26].

In the One Health surveillance characteristics domains, we focused on analyzing the One Health surveillance–related policies, legal instruments, governance, and leadership in Malawi, reflecting on the known characteristics of setting up and sustaining a One Health surveillance at the policy and institutional levels [2,9,10]. We further analyzed the stakeholders’ engagement processes and strategy for data integration and the technical infrastructure of the OHSP to see if it fulfills the One Health surveillance needs at the operational level [2,9].

**Table 1:** High-level analytic tool for health system resilience reflection and learning of the One Health Surveillance Platform establishment.

Domain	Thematic area	Establishment levels	Attributes and challenges
One Health surveillance characteristic	Policy and governance Sustainability Data integration Technical infrastructure	Policy level Institutional level Operational level	Policies, legal and operational framework Funding and scalability Data exchange across domains Hardware, software, and communication infrastructures
Addressing known DHIS2^a^ implementation challenges	Political, cultural, social, and structural infrastructure Appropriate data Workforce Education and training	Institutional level Operational level	Political challenges Stability Integrity of the health system or fragmentation Structural infrastructure Sufficient and qualified data Data quality Adequate technical support Comprehensive training programs
Health system resilience phases	Anticipation Preparation	Policy level Institutional level Operational level	Early detection and risk analysis Scenario planning Resource mobilization
Health system resilience attributes	Stakeholder Ecosystem Awareness Adaptive resilience Robustness	Policy level Institutional level Operational level	Stakeholder engagement Sectoral interaction Situation awareness Operational environment Adaptive structures Interoperability Coping strategies Withstanding challenges Capacity to handle increased demand Mobilization of essential resources
Health system resilience tools	Institutionalization	Policy level Institutional level Operational level	Capacity building System and infrastructure enhancement

^a^DHIS2: District Health Information Software 2.

Since the DHIS2 was chosen as the backbone technology for the OHSP, we applied the Addressing Known DHIS2 Implementation Challenges domain for analysis [25]. Among the 11 themes, we selected 4 relevant themes, focusing on analyzing the infrastructure issues, the data pipeline, and the workforce to provide technical support, as well as education and training to implement the OHSP, and synthesized the rest in other domains.

For the health system resilience phases and attributes, adaptive resilience was selected to assess the ability of the OHSP and development team to swiftly adjust to immediate challenges with flexibility while continuously evolving and improving based on past experiences and new information. Resilient digital health systems should be designed to adapt and maintain functionality, as well as enable smooth recovery and reintegration amid changing circumstances to accommodate new requirements, emerging threats, or evolving user needs. The practice of adaptability includes integrating new business requirements and new technologies, scaling up or down as necessary, and supporting interoperability with other systems [1,9,27,28].

Finally, since the OHSP is now institutionalized in Malawi and clearly stated in Malawi’s Health Sector Strategic Plan III 2023-2030 (HSSP III) [29], we applied the same reporting quality indicators, completeness, and timeliness, to assess the OHSP’s preliminary impact on weekly IDSR reporting at the national level [11]. Completeness was measured using the number of actual reports received against the number of expected reports from health facilities according to the health system level. At the same time, the timeliness of reporting was calculated based on the proportion of health facilities submitting surveillance reports on time to the district, with a national target of 80% for both indicators [11].

### Ethical Considerations

This study was approved by the Malawi National Health Science Research Committee with approval number 16/4/1563 and the Regional Ethics Committee, Regionale Komiteer For Medisinsk Of Helsefaglig Forskningsetikk sør-øst, with approval number 2015/2441/REK sør-øst A for its ethical conduct as part of the first author’s PhD study. The establishment of the OHSP was conducted under the supervision of the MoH to strengthen the country’s surveillance system in accordance with the relevant guidelines and regulations. All key informants were informed and obtained their verbal consent to provide information. The policies for analysis are publicly available. The meeting minutes from focus group meetings and key informant interviews, and field notes are kept safely by the first author and restricted access unless approval is obtained from the MoH. PHIM and the Digital Health Division of the MoH keep the technical documentation and digital health products. No compensation or reimbursement was provided to the meeting participants and key informants.

## Results

The observable implementation activities and outputs, including the establishment, implementation, and experience of the OHSP with the COVID-19 pandemic shock, and the post–COVID-19 pandemic impact on the IDSR reporting, are presented in this section based on the ADR method.

### Constitution of the OHSP Development Team

At the beginning of the study, the first author, TSJW, fulfilled his roles as an ADR researcher and practitioner in his capacity and was deeply immersed in the country’s digital health development context. He facilitated the constitution of the OHSP core team with the MoH leadership. The OHSP core team (n=12) comprises 2 government officials responsible for the nation’s IDSR system, 3 epidemiology unit and institution leaders at the PHIM, 5 digital health and IT staff from the Digital Health Division and Information Communication Technology Division of the MoH, a coordination officer, and a technical advisor. TSJW, the first author and technical advisor who was involved in the 2017 IDSR performance assessment, executed eIDSR pilots and provided technical inputs and guidance on developing the OHSP throughout the BIE stages. The extended OHSP development team consists of government technical officers from different sectors, in their role as the IHR focal person in the respective departments. The OHSP core team inherited technical officers supported by partners and sustained the institutional memories within the government from the previous eIDSR pilot projects by integrating them into the OHSP core team, and started working on establishing the OHSP technical solutions in 2019.

### Problem Formulation Stage

The establishment of the OHSP to support the eIDSR function was inspired by surveillance practices in the country and international recommendations, aligning with the third edition of the IHR 2005. The initial problems of the IDSR system were identified, highlighting the lack of technological solutions for weekly IDSR reporting. In order to tackle this problem, the first author, TSJW, continuously engaged the MoH and development partners to strengthen the IDSR system with 2 eIDSR pilots conducted from 2015 to 2018.

**Figure 1:**
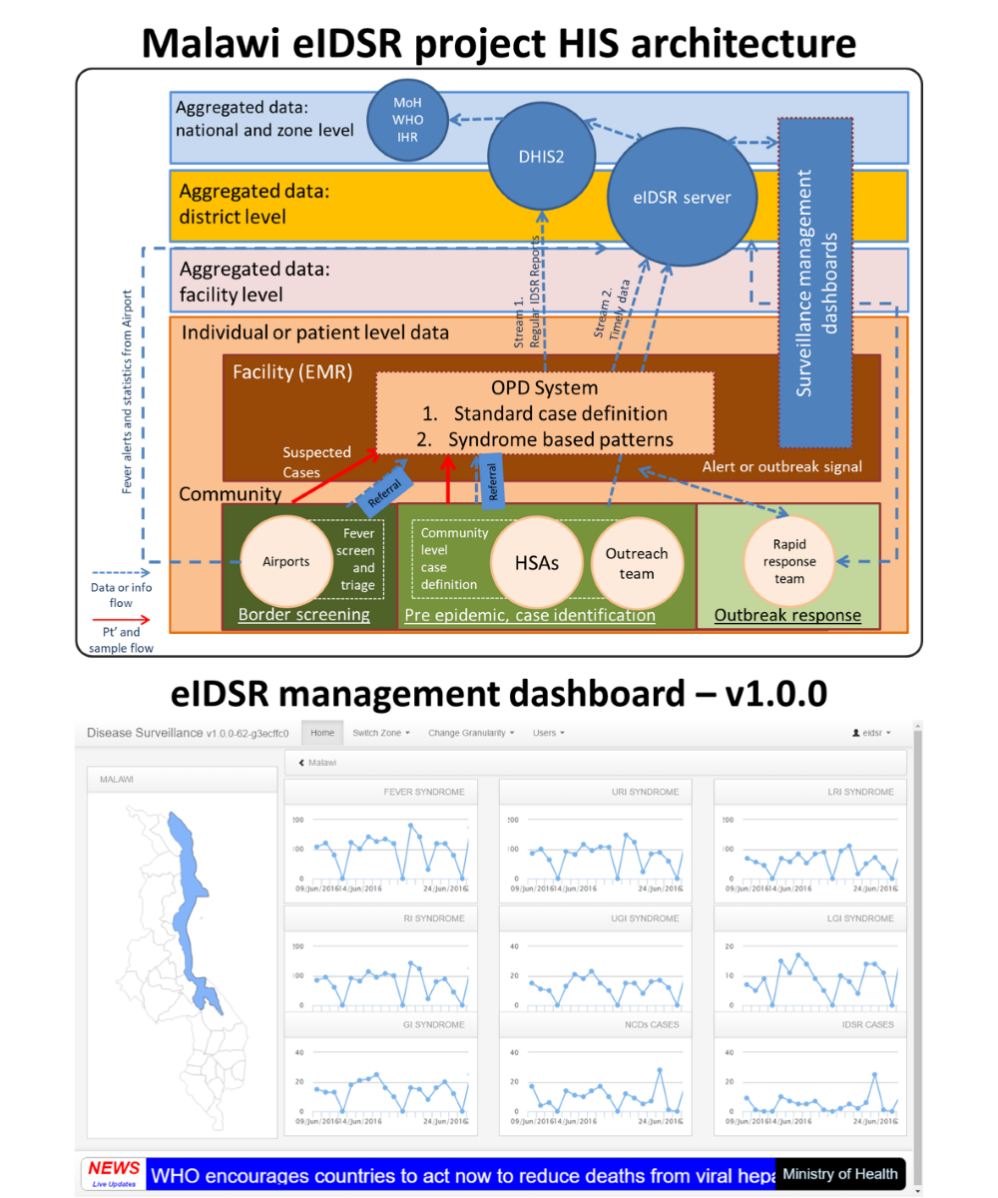
Preliminary electronic integrated disease surveillance and response (eIDSR) architecture and dashboard concept of the pilot in 2015. DHIS2: District Health Information Software 2; EMR: electronic medical record; HSA: health surveillance assistant; IHR: International Health Regulations; MoH: Ministry of Health; OPD: outpatient department, Pt’: patient; WHO: World Health Organization.

The World Bank supported the first pilot for the post-Ebola era with the aim of developing and implementing an eIDSR system at all levels of the health system in 2015. The following three main activities were conducted: (1) development of a comprehensive eIDSR system architecture and system logical design, (2) software development, and (3) system deployment and field support. During the first eIDSR pilot, we managed to develop the architecture design, establish a border health fever screening system, and develop the IDSR immediate notifiable disease reporting and syndromic reporting automated functions in the outpatient module of a partner-funded electronic medical record system (EMRS). The eIDSR architecture design used the organizational architecture level to guide respective information product developments and information flow; at the same time, the dashboard was designed to enable monitoring of the diseases and syndromic patterns of the outpatient visits together with a geographic information system and global health news Really Simple Syndication feeds function, as shown in Figure 1.

The following two main problems were identified based on the eIDSR pilot implementation results:

1. Sustainability: most of the project team members were dismissed after the project, leaving a minimal capacity within the MoH to maintain the developed systems. The management dashboard and eIDSR central database were designed in-house without the full transition of the source codes to the ministry, and the eIDSR functions in the EMRS were removed after the pilot due to a lack of funding for continuous support.
2. Technical infrastructure: the eIDSR pilot at the facility level was implemented in 59 health facilities with EMRS; however, it covered only 3.2% (59/1849) of the total public health facilities in the country at that time. By the time the pilot ended, the eIDSR pilot also did not cover the whole country for the needed periodic IDSR reporting (weekly and monthly) and community-level case detection.

In order to continue the eIDSR enhancement, the MoH obtained support from the United Nations International Children’s Emergency Fund (UNICEF) to pilot the World Health Organization (WHO) Argus solution and facilitate system integration for the country in mid-2017. The UNICEF support was to address the need for a timely weekly IDSR report using the technology. The solution was built and deployed to 220 health care workers for weekly IDSR reporting. However, sustainability and technical infrastructure challenges remained, with no subsequent financial resources and inadequate coverage of the facilities. During the pilot, an additional need was identified to have interoperability between the WHO-Argus solution and the nation’s HMIS, which uses DHIS2 technology. The 2 pilots provided lessons for the subsequent establishment of the OHSP. The prevention of the hippopotamus’ original anthrax outbreak in late 2018 inspired the MoH to decide to build a suitable digital system to accommodate the One Health surveillance needs and serve the eIDSR functions with the DHIS2 as the technological backbone support.

### BIE Cycles

#### Pre–COVID-19 Pandemic BIE Cycle

The OHSP establishment formally started in September 2019. Based on the pilot experience, the OHSP core team identified sustainability and technical infrastructure as the 2 key problems to be addressed in the forthcoming OHSP. In addition, previous core team members studied IDSR implementation gaps and community-level surveillance barriers in relation to technology for timely reporting; inadequate training, limited resources, and health-seeking behaviors affecting surveillance capability were also taken into consideration when the team started the OHSP establishment, as summarized in Textbox 1.

**Problems identified from the Malawi electronic integrated disease surveillance and response (IDSR) pilots and previous studies during 2015-2019.**

**Sustainability**

- Organizational sustainability
  Lack of coordination leading to duplication of efforts
  Lack of engagement from key personnel
  Unavailability of key Ministry of Health (MoH) staff
  Coordination issues for systems deployment
  Conflicting project schedules with some implementing partners
- Human resource sustainability
  User non-compliance with system usage
  Technical staff were dismissed without a full transition
  Inadequate technical personnel in the MoH to continue the development and maintenance
- Financial sustainability
  Unforeseen communication expenses
  Cash flow issues are delaying hardware procurement
  Lack of financial resources for scale-up and sustainability

**Technical Infrastructure**

- Incomplete functionality for the full IDSR system needs, including the community-level surveillance
- Low coverage of the solution
- Extended power outages are affecting deployment
- Connectivity issues affecting system functionality
- Labor-intensive setup process for the terminal device (smartphone)
- Congestion and delivery failures of the SMS service

The OHSP core team initiated the BIE stage to address organizational sustainability issues by reviewing the organizational structure across One Health domains, government documents, and literature to inform the design of the OHSP organizational architecture to accommodate One Health surveillance’s needs and the IDSR weekly electronic reporting function. Addressing the coordination and leadership issue was the first step. The core team conducted technical meetings with MoH leadership and learned that the One Health task force was formed during the anthrax response. Through these iterative meetings, we transparently communicated the governance concerns and affirmed that the ownership and leadership of the OHSP belong to PHIM within the MoH. PHIM’s leadership then led the core team to further engage with stakeholders to contribute to the architecture design of the OHSP. The OHSP core team began engaging with the animal and environmental sectors, with the animal health domain, represented by the Ministry of Agriculture, being fully consulted. While for the environmental domain, due to its vast fields, the team decided to engage further in the next phase of the OHSP implementation after the consultation with relevant stakeholders.

**Figure 2:**
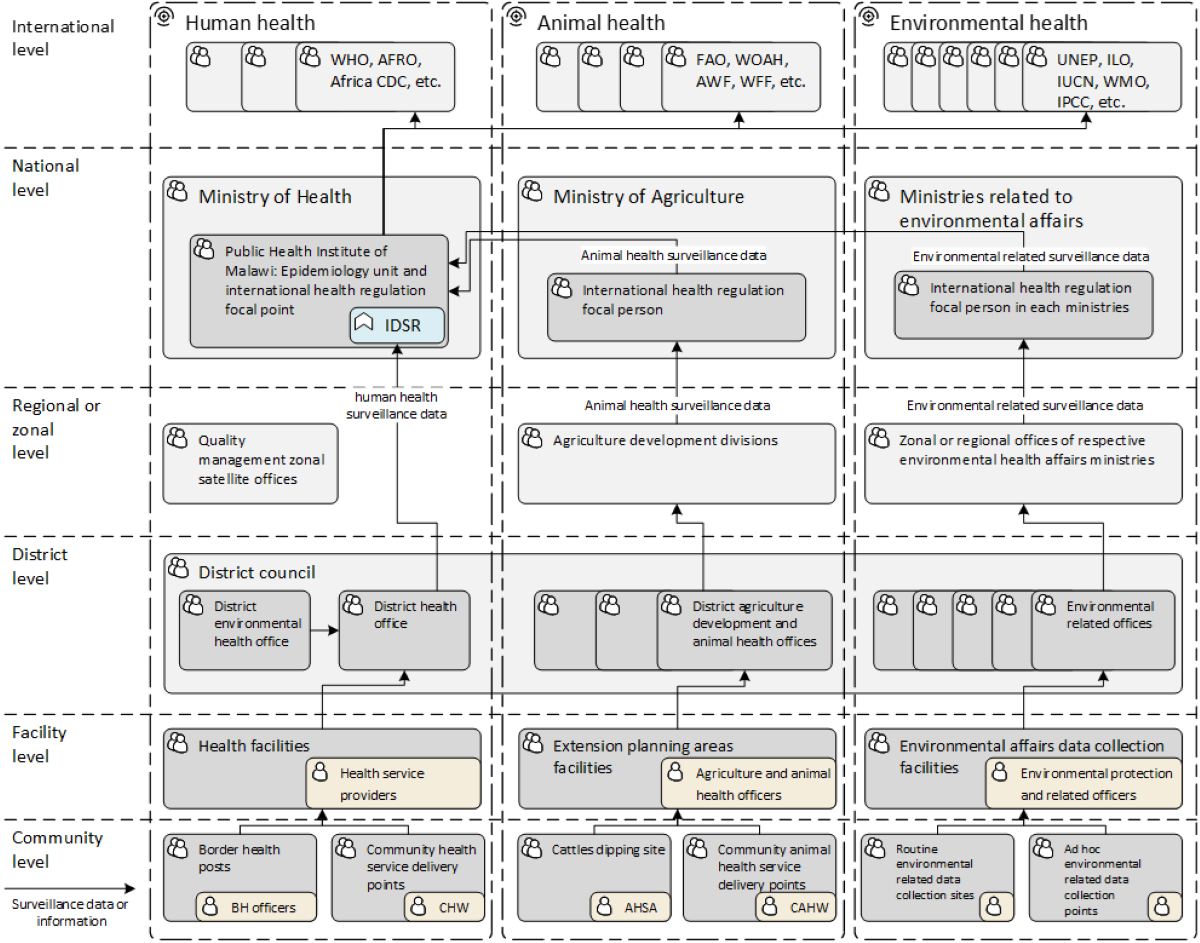
High-level business process architecture of the One Health Surveillance Platform in Malawi, 2019. AFRO: World Health Organization Regional Office for Africa; AHSA: animal health surveillance assistant; AWF: African Wildlife Foundation; BH: border health; CAHW: community animal health worker; CDC: Centers for Disease Control and Prevention; CHW: community health worker; FAO: Food and Agriculture Organization; IDSR: integrated disease surveillance and response; ILO: International Labour Organization; IPCC: Intergovernmental Panel on Climate Change; IUCN: International Union for Conservation of Nature; UNEP: United Nations Environment Programme; WFF: World Wildlife Fund; WHO: World Health Organization; WMO: World Meteorological Organization; WOAH: World Organisation for Animal Health.

The core team developed a high-level business process architecture of the OHSP (Figure 2), and aligned information flows based on the identified organizational units, surveillance personnel, and studied data collection tools. The hierarchy of the surveillance units is similar across domains but still shows differences at the subnational level across the 3 One Health domains, as listed in Table 2.

**Table 2:** Organizational units mapping between the 3 One Health domains in the context of Malawi.

Administrative level	Human health	Animal health	Environmental health
National level	Ministry of Health	Ministry of Agriculture	Ministries of Water and Sanitation, Natural Resources and Climate Change, Mining, Labour, and Land
Subnational level: region, zone, or division	3 regions, 5 health zones	8 agricultural development divisions	Different from each ministry
District level	29 health districts with district health offices	28 administrative districts with district agricultural development offices	28 administrative districts
Facility level	Central hospitals District hospitals Rural and community hospitals Health centers	Extension planning areas and sections where agriculture extension development officers work	Different from each ministry
Community level	Health post Village clinic Outreach clinic Village health committees (operated by community health workers)	Area development committees Village development committees (operated by animal health surveillance assistants	Different from each ministry

Upon stakeholder engagement, we noticed that only the human health surveillance system had standardized reporting and data collection tools using IDSR technical guidelines. Animal health surveillance has a reporting structure, but the standardized reporting forms and tools have yet to be updated and made available. Environmental health surveillance encompasses an even larger degree of complexity, including climate change, weather forecasts, air and water quality monitoring, waste management, pollution incident surveillance, occupational hazards, and risk surveillance.

After the high-level business process architecture was developed, the core team focused on establishing the main data repository to enable facility-level periodic IDSR reporting and to prepare for integrating animal and environmental health surveillance data pipelines in the next phase. The core team developed a strategy to mobilize future surveillance-related investment for human resource retention and to continue the enhancement of the OHSP to anchor and align the One Health surveillance architecture and existing solutions. At the beginning of the OHSP establishment, UNICEF supported the initial OHSP development and committed to initial implementation covering 48.3% (14/29) of the country’s health districts.

Once the high-level OHSP architectures were designed, the core team started working on the technical infrastructure for the establishment of the OHSP. Technical factors were considered to favor collaboration and integration of data from different domains, including DHIS2, as an existing national DHIS2-enabled digital health infrastructure used in HMIS, and an interoperability layer (IL), as an existing national information exchange infrastructure, to address these needs.

**Figure 3:**
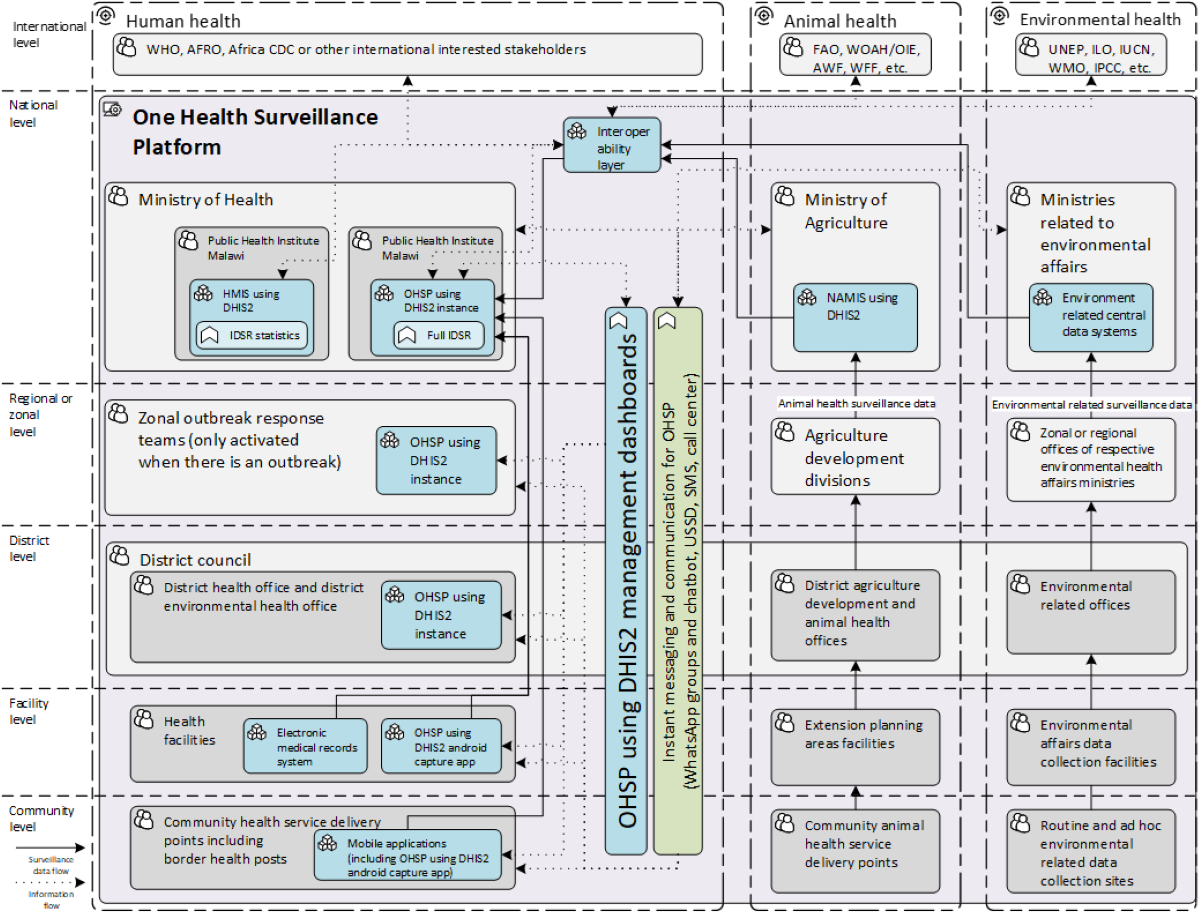
Technical architecture of the One Health Surveillance Platform (OHSP) after the transdisciplinary translation from public health needs to programmable digital health solutions in Malawi, 2019. AFRO: World Health Organization Regional Office for Africa; AWF: African Wildlife Foundation; CDC: Centers for Disease Control and Prevention; DHIS2: District Health Information Software 2; FAO: Food and Agriculture Organization; HMIS: health management information system; IDSR: integrated disease surveillance and response; ILO: International Labour Organization; IPCC: Intergovernmental Panel on Climate Change; IUCN: International Union for Conservation of Nature; NAMIS: National Agriculture Management Information System; UNEP: United Nations Environment Programme; USSD: unstructured supplementary service data; WFF: World Wildlife Fund; WHO: World Health Organization; WMO: World Meteorological Organization; WOAH: World Organisation for Animal Health.

The core team started the development of the OHSP in November 2019. In terms of roles, the technical staff were responsible for software development and deployment. The first author worked as a technical advisor serving leading and mediating functions in the communication between different stakeholders, including the requirements and limitations from different disciplines to the technical staff. Using a transdisciplinary approach, he translated the business process, information flow, and available tools into programmable technical requirements for developers. After the technical execution, the core team designed the technical architecture for the OHSP, as shown in Figure 3.

Based on the architectural design, the core team technical staff applied the Agile method, executing Scrum sprints to build the OHSP digital products. An instant messaging and communication collaboration mechanism was established and included in the OHSP. The One Health forum WhatsApp group was created for all IHR focal persons from different ministries to collaborate on health emergency preparedness and responses for real-time communications during the 2018-2019 anthrax outbreak response and was managed by the MoH. The functional One Health forum WhatsApp group served as one of the digital solutions in the OHSP, providing alerts to the group members. Similar WhatsApp groups have existed to facilitate IDSR coordination and collaboration since 2018. The experts in the One Health forum WhatsApp group anticipated that COVID-19 would become a great threat to the nation’s public health in mid-January 2020. The signal was provided to the core team for the team to focus on leveraging available infrastructure resources and reusing technologies from previous eIDSR pilots to develop the following three DHIS2-related digital products: (1) a national DHIS2 instance for the OHSP, (2) a DHIS2 Android Capture app for OHSP, and (3) an interoperability mediator between HMIS and OHSP.

The primary focus during January 2020 was setting up the national DHIS2 instance, especially the metadata design for weekly, monthly, and quarterly IDSR reporting. The core team paid attention to building the OHSP with integration and interoperability infrastructure, especially in relation to the HMIS. The IDSR periodic report and case-based surveillance form were standardized to provide appropriate data and replicated on the OHSP to match those in the HMIS to maintain data integrity. The integration was done by first matching the data element IDs, including the data field IDs and the dataset IDs. The organizational unit IDs were mapped and aligned with national standards using a master health facility registry. The team cloned existing facility mappings and data element mapping identification from the existing HMIS instance and registered the OHSP as a client in the nation’s IL so that it could recognize the OHSP instance as a source for data exchange.

Technical solutions were applied to ensure graceful degradation and seamless reintegration, including the DHIS2 features of PostgreSQL (PostgreSQL Global Development Group) performance tuning to improve query performance and scalability. The MoH-owned Proxmox (Proxmox Server Solutions GmbH) cluster was created to tolerate server downtime without affecting user access through multiple web servers. Load balancing within Nginx (F5 Inc) was configured to distribute load evenly across server instances and to detect any unavailable instances and reroute traffic as needed. The core team applied the “sticky sessions” solution of the DHIS2 to help maintain user session continuity by routing requests from the same client to the same server. The core team configured daily data backups of the OHSP to ensure data restoration capabilities. Fire and water safety controls and a continuous power backup supply at the national digital health server room were applied for the OHSP to operate continuously with minimal downtime. For security, the OHSP DHIS2-related products used password protection and controlled access.

In February 2020, the team concentrated on ensuring HMIS-OHSP interoperability. When building the interoperability, an initial script was created to directly transfer the IDSR data from the HMIS instance into the OHSP. However, when running the script, there was a time-out due to the huge payload that was being sent from HMIS to OHSP; hence, the team decided to use the IL for chunking functionality. Process-wide, the IDSR periodic reporting process was not standardized; some facilities reported in HMIS, while some reported in the OHSP. The MoH leadership addressed the issue by disseminating an internal memo to regulate all surveillance data to be entered in OHSP. The core upgraded the HMIS and OHSP instance, so the payload was channeled through the IL to use the chunking of the payload when sending it from the OHSP to the HMIS.

Simultaneously, the team worked on the integration and user interface design of the DHIS2 Android Capture app to interact with the OHSP instance for data collection and reporting. Also, the team worked on obtaining additional public IP addresses with the government domain for OHSP to facilitate Secure Sockets Layer acquisition and deployment of the solutions through the internet. By March 2020, OHSP was tested by PHIM and was fully ready for implementation to support Malawi’s surveillance and response needs.

#### During the COVID-19 Pandemic BIE Cycle

Right before the core team was ready to deploy the OHSP products, new requests emerged as the country faced the impending threats from the COVID-19 pandemic. According to the original plan, the first release of the OHSP products was to facilitate timely IDSR periodic aggregated data reports. However, with the emergency, the MoH and the national COVID-19 response team at the emergency operation center raised requests and digital health solutions needs, which required additional functions, integration, and interoperability between the OHSP products and other digital health solutions.

The COVID-19 response emergency operation center was formed according to the Africa Centres for Disease Control and Prevention recommendations in April 2020. The first author, TSJW, was appointed acting manager at the COVID-19 emergency operation center that same month, supporting response activities while continuing to serve on the core team for OHSP development. The core team responded to the requests raised and prioritized them according to the consensus and guidance from the government by leveraging available digital solutions and the IL. The case-based surveillance form of the IDSR system in OHSP was harmonized and standardized with various stakeholders’ expectations and updated with COVID-19 conditions in May 2020. Additional forms for border health declaration and contact tracing were developed and added to the OHSP during May-June 2020. The validation rules for mandatory data entry were programmed in the OHSP to ensure data quality at the same time. After the new features of OHSP products were developed, they were included in the new national emergency preparedness and response plans to support the response from May 2020.

The core team applied the agreed strategy to mobilize resources for the national scale-up of OHSP instead of only implementing it in 14 districts when funders and partners showed interest in investment resources for pandemic response. Through the government leadership and collaboration mechanism, multiple funders accepted the advocacy at the beginning of the COVID-19 pandemic, and the core team secured access to additional technical infrastructure resources, especially the server, terminal devices (tablets), and reverse billing approach of connectivity, for deploying the OHSP products countrywide since June 2020. However, the team was unable to mobilize adequate resources for comprehensive redundancy and backup servers of OHSP DHIS2-related products till the end of the pandemic in May 2024.

During the national rollout, all partners followed the MoH leadership to provide standardized training, follow-up supervision, and refresher training. A training program for OHSP products users was designed and implemented comprehensively with user manuals, standardized presentations, a deployment checklist, and detailed schedules, covering computer literacy from the beginner level. A centralized digital health helpdesk at the Digital Health Division was established to provide support to OHSP users. The core team members received further education and training for their DHIS2-related capacity building through online and in-person participation in DHIS2 academies from 2021 to 2023.

A national OHSP enhancement plan was developed to institutionalize OHSP, and a dedicated technical working group was established to facilitate One Health surveillance and regular updates and the One Health bulletin at PHIM in 2023. A comprehensive capacity-building plan and a system infrastructure enhancement plan of OHSP were developed to integrate animal health and environmental health data pipelines in 2024. After the deployment, the OHSP core team has constantly collected the needs and feedback from relevant stakeholders through the established collaboration mechanism. Since then, the OHSP digital products have been sustained and continue to be used for IDSR reporting to date.

#### Post–COVID-19 Pandemic OHSP’s Impact on IDSR Reporting

**Figure 4:**
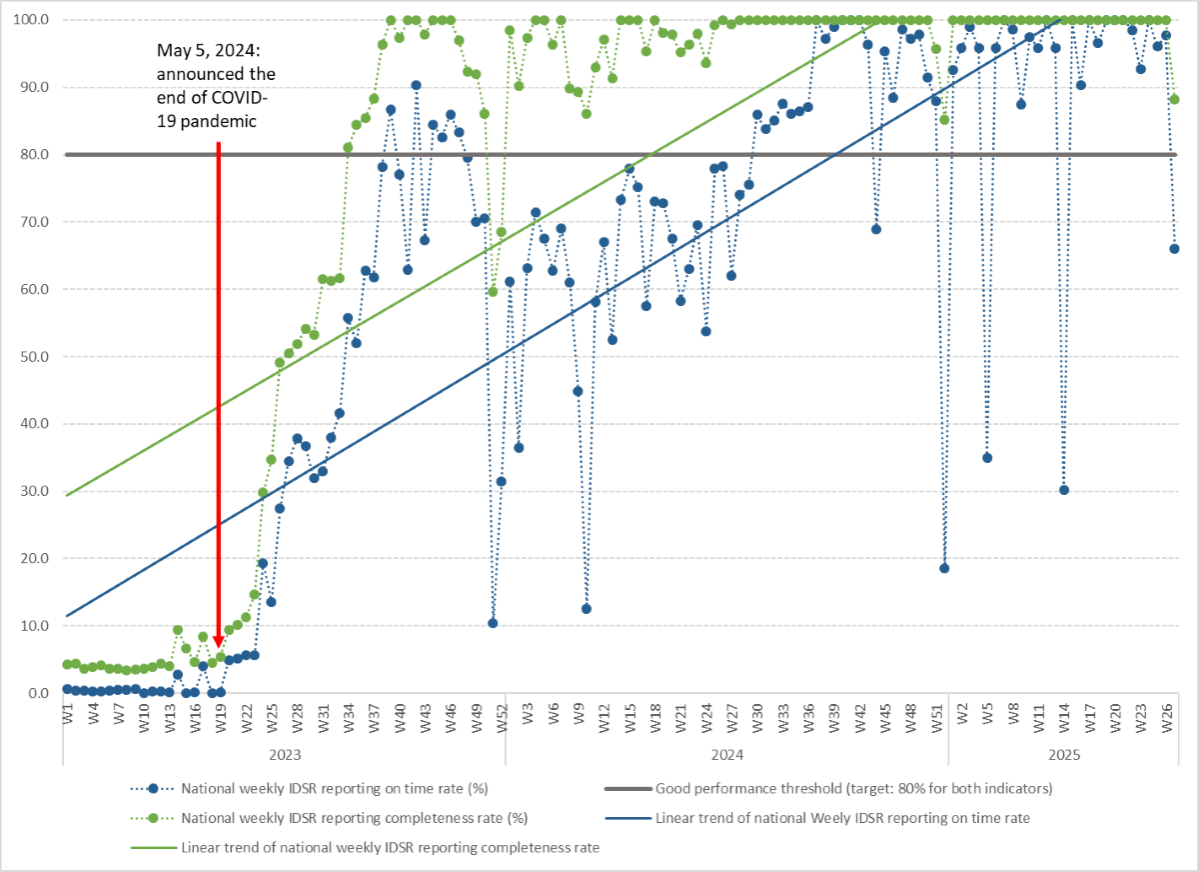
The integrated disease surveillance and response (IDSR) weekly reports completeness and timeliness indicators from January 2, 2023, (epidemiological week 1) to June 29, 2025, (epidemiological week 26) in Malawi.

The OHSP became the main digital data repository for all IDSR surveillance periodic reports after the pandemic. The interoperability mediator is functioning with the MoH as the custodian, ready for reuse and tapping into other One Health surveillance domains’ data. The One Health forum WhatsApp group is still functioning to facilitate the ongoing One Health surveillance activities. The completeness and timeliness of IDSR periodic reports’ data quality indicators were defined according to the national guideline and configured in the OHSP’s national DHIS2 instance. The MoH continuously provides appropriate feedback on data quality issues through the dashboards, bulletins, and WhatsApp groups to reporting facilities to ensure data quality, with the OHSP core team maintained within the MoH governance structure. The OHSP dashboards developed provide functionality to monitor the epidemiological patterns of notifiable diseases routinely for outbreak detection. As a result, the weekly IDSR reporting transformed from nonexistence in 2015 to a state where the nation reached 97.8% and 74.5% reporting quality for completeness and timeliness, respectively, in 2024. The secular trends and weekly IDSR reporting rate quality are illustrated in Figure 4.

## Discussion

### Summary of Main Findings

This study aims to report on the establishment of the OHSP in Malawi and how an adaptive digital health solution contributed to strengthening and impacting the country’s IDSR system during the COVID-19 pandemic and beyond the pandemic. Our findings show that Malawi successfully established a government-led digital tools–aided OHSP right before the COVID-19 pandemic, building on the ongoing effort and further strengthening the country’s IDSR system. The OHSP was built corresponding to the characteristics of One Health surveillance systems across policy, institutional, and operational levels [1,2]. Amid the COVID-19 pandemic, the MoH evidently used the OHSP to mitigate the spread and impact of the pandemic [21], and the weekly IDSR reporting improved significantly after the pandemic, from zero reporting in 2015 [11] to 97.8% completeness and 74.5% timeliness by 2024, while PHIM is currently undertaking a full impact assessment of the OHSP.

Findings from this study indicate that the alignment of the national health policies and the establishment of a formal coordination body with government leadership were essential to the One Health surveillance governance. The government-owned and government-led strategy was useful for communicating with all potential funders to invest in the OHSP and align with the government’s direction.

The developed holistic OHSP architecture guided the data and information integration design, especially on organizational units and reporting tools standardization. The mapping of the corresponding organizational units with the human health system hierarchy at each administrative level for animal and environmental domains, and having standardized data elements, was a complex task. Considering the complexity, the OHSP core team sensitized all stakeholders engaged in helping with the proper mapping of the units and understanding of the importance of the data standards for future One Health surveillance data exchange and interoperability.

The reuse of the eIDSR pilots’ hardware and available digital solutions provided adequate hardware, software, and communication infrastructure for the initial establishment of the OHSP and accelerated in responding to the COVID-19 pandemic needs. Several known DHIS2 implementation challenges were addressed during the establishment of the OHSP. The OHSP anticipation and adaptive resilience abilities were observed in the study. A detailed discussion, comparison, and contrast of findings with existing literature is followed.

### Interpretations and Implications of Lessons Learnt

#### One Health Surveillance Characteristics

Our findings were reflected based on four thematic areas using the analytic tool developed (Table 1) under the One Health surveillance characteristics domain, corresponding to the policy, institutional, and operations levels according to the One Health surveillance framework developed by Bordier et al [2].

#### Policy and Governance at the Policy and Institutional Levels

As known challenges, robust policy frameworks and governance structures are essential factors toward a successfully integrated One Health surveillance system [2,8-10]. The third edition of the IHR 2005 [30] was the main international legal instrument used to justify the One Health surveillance initiative in Malawi. The Joint External Evaluation conducted in Malawi in 2019 highlights the importance of operationalizing and strengthening the One Health platform, improving surveillance systems, and enhancing the data-sharing platform [15], providing an operational framework for Malawi to take action.

The Public Health Act (1948) [31] and the Control and Diseases of Animals Act (1967) [32] provide legal frameworks for disease surveillance, reporting, and control in both human and animal health sectors in Malawi. The Environment Management Act (2017) [33] complements the One Health approach by promoting environmental protection, monitoring, reporting, and public participation in decision-making processes. Malawi’s health sector policy, especially the HSSP III [34], highlights the adoption of the One Health approach and OHSP, particularly in areas of antimicrobial resistance and climate change, echoing the One Health surveillance focuses found by Bordier et al [2] and Malawi’s emerging needs [17]. At present, the OHSP is institutionalized at PHIM in Malawi to strengthen the IDSR reporting quality, avoiding potential abandonment of the digital health initiative after the COVID-19 temporary use [29]. The developed OHSP enhancement plan and HSSP III [34] guide the gradual integration of different One Health domain data pipelines using the OHSP digital tools. These are useful legal instruments that solidify the government-led coordination mechanism at the policy and institutional level, as advocated in relevant literature [2,5,6], and may be applied in other settings.

Concerning the governance and operation of collaborative surveillance across different sectors, the political will is essential in establishing a One Health surveillance system and may foster its success [2,9,10,35]. Our findings clearly indicated that Malawi showed strong political will, solidified soon after the successful anthrax outbreak response, and gained momentum from the resilient response to the COVID-19 pandemic. The 2 instances affirmed the leadership role of PHIM and strengthened the commitment of the government to take the same One Health approach for future health emergencies. As advocated, government-owned and led digital health interventions offer an opportunity to strengthen health systems and attain health-related goals on the continent [36]. The reinforced government commitment to OHSP’s development had effectively reduced the creation of “silos” in the health surveillance system. Political will and leadership are the two key enabling factors contributing to the sustainable operation of the One Health surveillance system, echoing the findings from Vietnam and Australia’s experience [10,35]. Coordinated implementation with government leadership maintained continuity and OHSP ownership, further ensuring financial and organizational sustainability in Malawi at the institutional level [2,26].

#### Sustainability for All Levels

The affirmation of the One Health leadership, established coordination mechanisms, and policy alignment secured the organizational sustainability of Malawi’s One Health surveillance initiatives, reflecting the advocacies from others [2,9,10]. The government officials at the forefront led the implementation and delivery of the OHSP digital products to avoid district and facility health care workers conceiving the OHSP as an external system. This approach further reduced operational costs and silo creation, ensuring long-term sustainability as argued by academics in the same field [2,8,10,35]. Meanwhile, the reuse of technology strategy with open-source digital solutions, such as the global DHIS2 and Open Health Information Exchange communities [25,37], provides technical advancement and long-term software sustainability, coupled with the ongoing maintenance by the core team, making the OHSP a resilient information system [26].

Concerning financial sustainability, the strategy of advocating and lobbying for surveillance-related investment to anchor and align the One Health surveillance architecture was a useful approach for the OHSP to scale up nationwide, with the COVID-19 pandemic providing the MoH with an opportunity to align and direct partners’ investment to support it. Government ownership and leadership of the OHSP avoid the potential interruption, silos, or hiccup of funding availability, responding to the challenges observed and concepts reported before [2,5,9,10,35]. The OHSP, as a government system, with or without external financial support, requires all civil servants to commit to making it work.

#### Data Integration at the Operational Level

Most of the One Health surveillance systems in action are focused on zoonotic diseases [38,39] for data to be integrated with human surveillance. The established OHSP can swiftly connect with animal and environmental health surveillance data with its adaptability. In fact, the recent development of the National Agriculture Management Information System (NAMIS) in Malawi also uses the same DHIS2 technology to support the Ministry of Agriculture in improving livestock production, enhancing herd health, and addressing transboundary animal diseases [40]. The OHSP can provide its affordance to interoperate with NAMIS for surveillance needs. Environmental health, by the nature of its vast domain, has more work to do. Therefore, the OHSP enhancement plan focuses on climate change in alignment with HSSP III can be supported by the same DHIS2 solutions [41]. However, as per our findings, the standardization of organizational units to cover all One Health surveillance domains will be a key issue that needs to be addressed, ideally using a similar master facility registry for animal and environmental surveillance sites.

#### Technical Infrastructure Reuse at the Operational Level

The functional OHSP, as an effective information system, addressed Malawi’s Joint External Evaluation report issues and lack of weekly IDSR reporting by operationalizing and strengthening the One Health platform [11,15]. The high-level architecture, inspired by the concept of the integrated health information system architecture [42] and its alignment with IDSR processes, supported the design of OHSP and the strategy to reuse the existing infrastructure. Although the collaboration with stakeholders during the COVID-19 pandemic secured adequate technical infrastructure for the nationwide OHSP scale-up; however, the current collaborative modalities and technical support mechanisms are still limited in the human health domain, similar to other settings’ One Health surveillance progress [38,43,44], where investment will be needed in other domains.

Digital health tools have proven instrumental in improving resilience and maintaining essential health services during crises [21]. However, the challenges noted in the infrastructure, particularly the lack of comprehensive redundancy and the limitations of the server room, point to the need for continuous investment, as highlighted by engineering resilience [26], to have a robust technical infrastructure to ensure OHSP’s scalability and reliability.

#### Corresponded to Known DHIS2 Implementation Challenges

Findings were mainly reflected and compared with Dehnavieh et al [25].

##### Political, Cultural, Social, and Structural Infrastructure

The legal instruments, MoH’s leadership, and institutionalization of the OHSP at PHIM addressed known politically related challenges [25]. However, as discussed earlier, sustaining stable financing for technical infrastructure remains a challenge.

##### Appropriate Data—Quality and Standards

The standardized IDSR data collected through the OHSP—including updates and additions introduced during the COVID-19 pandemic—along with validation rules for mandatory data entry, defined data quality indicators, data quality monitoring dashboards, and a weekly epidemiological bulletin ensured appropriate surveillance data supplies. These results directly addressed the challenges identified in Dehnavieh et al [2,25].

##### Workforce and Capacity Building

Malawi has a relatively adequate workforce for the development and implementation of OHSP DHIS2-related products, as evidenced by the constitution of the core team consisting of government officials and technical support officers from partner organizations who programmed the DHIS2 solutions locally. The OHSP enhancement plan, capacity-building plan, and online DHIS2 academies [45] addressed the human resource challenges [25].

#### Health System Resilience—Anticipation and Adaptive Resilience

Concerning the health system resilience, Malawi’s health emergency awareness capacity was enhanced through real-time communication using WhatsApp groups for timely information sharing and decision-making. The use of such a digital solution with the One Health approach corresponds to the Riyadh Declaration to improve preparedness and response to future pandemics [46]. However, although the MoH manages these WhatsApp groups, risks remain for intentional information leakage by group members.

The adaptability of the OHSP was evident in the rapid response to COVID-19, with adjustments made to the system based on emerging needs. This finding echoes the recent organizational resilience studies during COVID-19 about adaptation, innovation, capabilities, and the use of new technology [47]. The Agile software development method, a skilled and committed core team, and affordable digital tools made the success possible.

The result demonstrated that Malawi’s OHSP is a resilient system with high adaptability that supports the surveillance goals during the COVID-19 pandemic [21]. The OHSP core team consistently managed to build effective governance with strong government leadership to provide clear policy directions, which are essential for building resilient health systems [1,4]. The government’s strong leadership in the establishment of the OHSP paved the way for OHSP digital health solutions to be developed with resilience and rapidly scaled nationwide, echoing conclusions on Africa’s digital transformation [36].

### Remaining Gaps and Future Directions

The OHSP addressed some identified barriers to support the IDSR system [11]; however, the current functionality has yet to address the community-level surveillance gaps fully [48]. Event-based surveillance (EBS) is one of the recommended surveillance methods to support community-level surveillance, as stated in the recent IDSR technical guidelines [12]. Other countries’ EBS implementation experiences and practices encountered some challenges, including the trade-off between sensitivity and specificity, low awareness of EBS, and inadequate infrastructure [49,50]. The role of middle-layer actors in participating in community-level surveillance, community engagement, and additional digital solutions should be considered as the OHSP is enhanced to support the EBS in the country, not only for human health but also animal, including wild and domestic, and the surrounding environment as a whole [1,48,50]. Engagement with stakeholders using existing coordination mechanisms and applying the participatory approach should be encouraged for future OHSP enhancement [35]. Despite Malawi managing to address the OHSP establishment challenges through strategic planning and implementation, continuous capacity building, training, supervision, and retaining skilled personnel for the OHSP to be resilient still exist in Malawi with constrained resources, similar to the challenges that Tanzania encountered trying to establish a similar platform [51]. Mobilizing and lobbying resources to fully operationalize the OHSP enhancement plan, with highlighting multisectoral benefits as argued by Fasina et al [44], is the recommended direction.

### Limitations

Our study focused on the establishment experience of the OHSP in Malawi; despite its impact on the weekly IDSR reporting quality, its further use, linkage to other One Health domains, and its impact on the overall One Health surveillance are limited. Regarding the health system resilience, although the frameworks were developed [5,6], they are limited to emergency management and health care systems, with none specifically for One Health surveillance systems [26,52,53]. The synthesis analytic tool we developed for the OHSP establishment analysis can be used in similar settings for assessing the One Health surveillance system, despite the fact that our analysis was limited to the One Health surveillance system establishment stage; further use of the tool is encouraged.

### Conclusions

The success of Malawi’s OHSP establishment highlights the importance of government leadership and coordination in ensuring financial and organizational sustainability. Key findings informed the need for continuous capacity building, adaptable technical infrastructure, and enhanced community-level surveillance in the long run.

As global health systems continue to face emerging threats to respective contexts, Malawi’s experience demonstrates that a well-integrated, government-led digital OHSP can enhance resilience and preparedness to these health challenges. Amid the dynamic of the global development policy changes and complex human-animal-environmental interactions, findings from this study can be applied to broader audiences to enhance their surveillance and health systems in combating the pandemics to come.

Future investments, with emphasis on local leadership and domestic financial resources, in interoperability, infrastructure, and community engagement, will be essential for maximizing the impact of OHSP and similar initiatives worldwide.

## Appendix

Multimedia Appendix 1 Detailed analytic tool for health system resilience reflection and learning of the One Health Surveillance Platform establishment.

## Abbreviations

ADR: action design research
BIE: building, intervention, and evaluation
DHIS2: District Health Information Software 2
EBS: event-based surveillance
eIDSR: electronic integrated disease surveillance and response
EMRS: electronic medical record system
HMIS: health management information system
HSSP III: Health Sector Strategic Plan 2023-2030
IDSR: integrated disease surveillance and response
IHR: International Health Regulations
IL: interoperability layer
MoH: Ministry of Health
NAMIS: National Agriculture Management Information System
OHSP: One Health Surveillance Platform
PHIM: Public Health Institute of Malawi
UNICEF: United Nations International Children’s Emergency Fund
WHO: World Health Organization

## Data Availability

All meeting minutes and internal notes are available upon request. The One Health Surveillance Platform–related source codes and documentation, for public access according to the Ministry of Health regulations, are available in the following GitHub repositories: (1) Ministry of Health of Malawi in production services GitHub repository [54] and (2) Ministry of Health of Malawi Project-Based GitHub repository [55].
